# Handheld 6-Lead ECG for Early Detection of Acute Inferior Wall ST-T Segment Elevation Myocardial Infarction: HINT-MI Study Design and Rationale

**DOI:** 10.3390/medicina60071164

**Published:** 2024-07-18

**Authors:** Sodam Jung, In-Sook Kang, Sanghoon Shin, Choongki Kim, Junbeom Park

**Affiliations:** 1 Division of Cardiology, Ewha Womans University Mokdong Hospital, Ewha Womans University College of Medicine, Seoul 07985, Republic of Korea; cardioj@ewha.ac.kr (S.J.);; 2 Division of Cardiology, Ewha Womans University Seoul Hospital, Ewha Womans University College of Medicine, Seoul 07804, Republic of Korea

**Keywords:** ST elevation myocardial infarction, electrocardiogram, early diagnosis, mobile device, handheld device

## Abstract

*Background*: ST-T segment elevation myocardial infarction (STEMI) is a critical condition that requires rapid diagnosis and treatment. Recently, various ECG recording devices have been developed. In this study, we aim to determine the utility of a 6-lead handheld ECG recording device to shorten the time taken for the diagnosis of inferior wall STEMI. *Methods and Design*: HINT-MI is an investigator-derived, observational, prospective study that will evaluate the ability of a handheld 6-lead ECG device to diagnose acute inferior wall STEMI. Patients who have undergone coronary angiography for STEMI or for other reasons will be enrolled in the study. This study aims to evaluate sensitivity and specificity of a handheld 6-lead ECG device by the level of agreement with a standard 12-lead ECG for diagnosing inferior wall STEMI. Further, we will determine whether the use of the handheld device can reduce the time needed for reperfusion treatment through faster diagnosis. *Conclusions*: This study aims to investigate the feasibility of a handheld 6-lead ECG device for diagnosing inferior wall STEMI to reduce the time required to diagnose inferior wall STEMI and to allow timely treatment.

## 1. Introduction

ST-T segment elevation myocardial infarction (STEMI) is a critical condition that requires rapid diagnosis and treatment [1]. The European Society of Cardiology 2017 guideline and the American Heart Association guideline suggest time targets related to the diagnosis and treatment of STEMI [2,3]. The usual practice is to diagnose STEMI in patients with chest discomfort or other ischemic symptoms and new ST-segment elevations in two contiguous leads [4]. Electrocardiography (ECG) should be performed within 10 min of the first medical contact, and if STEMI is diagnosed, percutaneous coronary intervention (PCI) should be performed within 60 min (wire crossing) at a primary PCI center [2,3]. However, when obtaining a 12-lead ECG from a patient complaining of chest pain in the emergency room, owing to the large size of the machine, it takes 3–5 min to move it next to the patient and record the ECG.

Recently, various ECG monitoring devices have been developed to diagnose arrhythmia such as atrial fibrillation through long-term monitoring [5,6]. Several studies have attempted to obtain chest lead equivalents or diagnose STEMI using a single-lead handheld ECG recorder or a smartwatch and determine its correlation with a standard 12-lead ECG [7,8,9,10]. Moreover, the recent research focus has been to generate a 12-lead ECG from a single-lead ECG [11] or to predict STEMI using artificial intelligence [12]. However, studies related to diagnosis using a handheld 6-lead ECG have not yet been published.

The smartphone-based handheld 6-lead ECG recording device, Alivecor Kardia Mobile 6 L, has two front transducers and a back transducer. It contacts both the upper limbs and one lower limb (e.g., left iliac crest, thigh, knee, and lower abdomen) to obtain all limb leads (I, II, III, aVR, aVL, and aVF). Anterior wall or lateral wall STEMI generally requires a precordial lead ECG in addition to a limb lead ECG for diagnosis [4]. Inferior wall STEMI can be diagnosed using limb leads alone. The capability to diagnose inferior wall STEMI by recording an ECG for 30 s using a handheld ECG device could potentially expedite diagnosis compared to existing devices that require significant time to move or attach to a standard 12-lead ECG device (at least 3 to 5 min). The use of this handheld device is expected to shorten the time required for diagnosis and making treatment decisions for inferior wall STEMI.

This study aims to evaluate the level of agreement between the results of a handheld 6-lead ECG device and a standard 12-lead ECG in the diagnosis of inferior wall STEMI to determine whether the use of the handheld device can reduce the time needed for reperfusion treatment through faster diagnosis.

## 2. Materials and Methods

### 2.1. Study Design

Handheld 6-lead ECG device for the early detection of acute inferior wall ST-T segment elevation myocardial infarction (HINT-MI) is an investigator-derived, multicenter, observational prospective study to evaluate the ability of a handheld 6-lead ECG device to diagnose acute inferior wall STEMI (reg. no. clinicaltrials.gov NCT05777083). By comparing a 6-lead ECG using a handheld device and a 12-lead standard ECG in patients with various types of STEMI and those without STEMI, the diagnostic accuracy of the 6-lead handheld ECG device in inferior wall STEMI will be evaluated.

### 2.2. Primary Objectives

This study will investigate the sensitivity and specificity of a handheld six-lead ECG device compared to a 12-lead ECG for the diagnosis of acute inferior wall myocardial infarction.

### 2.3. Study Population

Two primary PCI centers in South Korea are participating in this study, representing full coverage of all levels of care, including state-of-the-art tests, monitoring, imaging equipment, the latest treatment regimens, access to specialists, and intensive care units. The study subjects will consist of two types of patients: (a) patients who will first visit the emergency room and be diagnosed with various types of STEMI by a 12-lead ECG and undergo primary PCI, and (b) patients without STEMI who will undergo coronary angiography for chest pain after visiting an outpatient clinic. All the patients enrolled in this study will provide their voluntary informed consent.

In the case of patients with all kinds of STEMI, a surface 12-lead ECG in the emergency room and coronary angiography results will be used to screen for STEMI. Patients initially diagnosed with STEMI by ECG but later confirmed not to have a myocardial infarction by additional tests (coronary angiography and serum cardiac markers) will be excluded from the study. The diagnosis of myocardial infarction will follow the criteria of the fourth universal definition of myocardial infarction [4]. Patients with severe complications of myocardial infarction such as cardiogenic shock, respiratory failure, or decreased consciousness will be also excluded.

### 2.4. Six-Lead Handheld ECG Recorder

**Figure 1:**
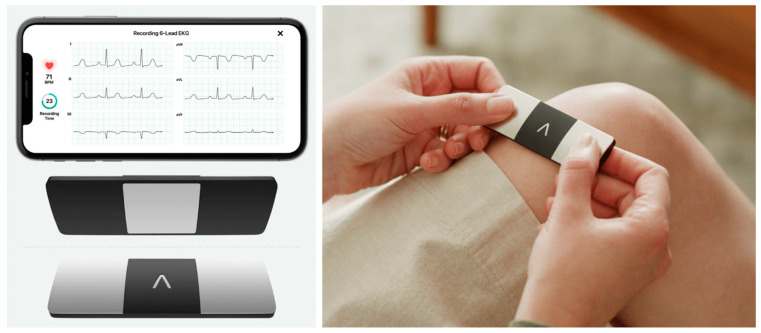
Handheld 6-lead ECG recording device in this study. The device is used with finger of right and left hand touching the respective electrode and a sample ECG rhythm is shown in a mobile phone display. Copyright with permission from Alivecor.

A recently developed wearable device for the detection of arrhythmia, a handheld 6-lead ECG recorder based on a smartphone (Kardia Mobile 6 L TM; Alivecor Inc., San Francisco, CA, USA), enables the patient to measure and transmit the ECG of the limb leads (Figure 1). It has been clinically validated for detecting atrial fibrillation in various populations. In our study, a handheld 6-lead ECG recorder will be used to record the limb leads. Access to the data is restricted to those authorized by institutional review board to handle data related to patient care and this study. The server to which the ECG is transmitted will be managed by the manufacturer. The device manufacturers had no role in the trial design, data accrual, or analysis.

### 2.5. ECG Recording

#### 2.5.1. Patients with STEMI

Among all patients with various types of STEMI who visit the emergency room and do not meet the exclusion criteria, an ECG using a 6-lead handheld device will be recorded, in addition to a basic 6-lead ECG monitor and a 12-lead ECG. In general, all patients with STEMI who visit the emergency room undergo a 12-lead ECG in the emergency room and then move to a catheterization laboratory for coronary angiography. In this study, the first 6-lead ECG by the handheld device will be recorded for 30 s in the supine position. To prevent any treatment delays, we will record the handheld ECG as part of the procedure preparation process, including the attachment of basic monitoring. If the first handheld recording is not available, it will be recorded before leaving the catheter lab.

During the procedure, a wired 6-lead ECG is typically attached to continuously monitor the patient’s condition. We will record this data for 30 s before and after the procedure. Finally, a 12-lead ECG will be recorded within 1 h after moving to the intensive care unit or emergency room, according to the general myocardial infarction treatment plan.

#### 2.5.2. Subjects Receiving CAG without STEMI

**Figure 2:**
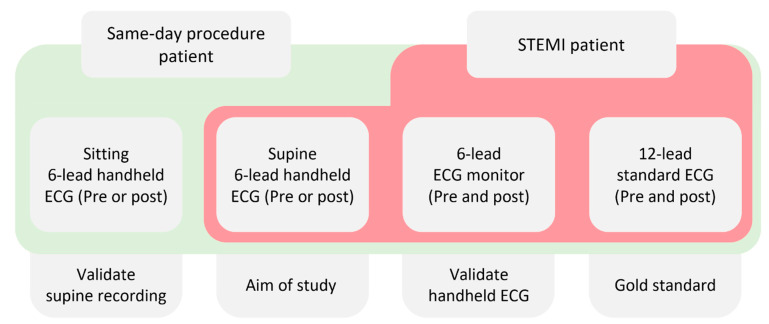
Schematic diagram of the study design. ECG = electrocardiogram; STEMI = ST elevation myocardial infarction.

Data will be collected from patients who do not meet the exclusion criteria among those without STEMI who visit the hospital for chest pain and are hospitalized to undergo coronary angiography. In general, for patients admitted to the hospital for coronary angiography via an outpatient clinic, a standard 12-lead ECG is performed in the ward. While preparing for the procedure, basic monitoring (such as 6-lead ECG or pulse oximetry) will be performed immediately upon arrival of the patient in the angiography room, and a handheld 6-lead ECG will be recorded for 30 s in the supine and sitting position, before or after the procedure. At the same time, the data from the wired 6-lead ECG continuously monitoring the patient will be stored and collected (Figure 2).

**Figure 3:**
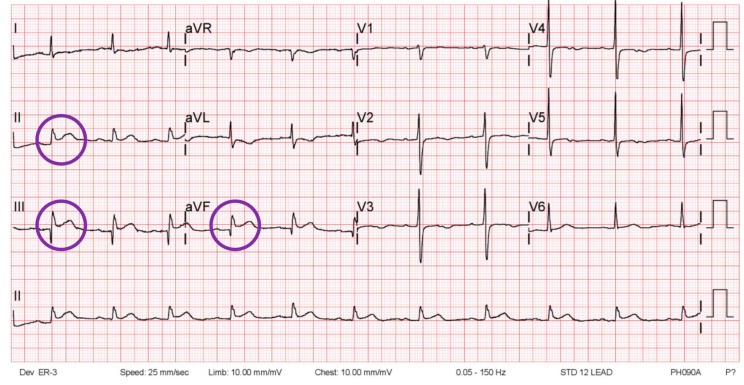
An example of 1 mm elevation of ST segment (circles) in II, III, aVF leads of inferior STEMI patient. STEMI = ST elevation myocardial infarction.

A standard 12-lead ECG, 6-lead ECG monitor recording, and handheld 6-lead ECG data will be reviewed in a blinded manner by cardiologists with anonymized patient information. The ECG results will be matched with the case number and the sensitivity and specificity of the handheld 6-lead ECG performed before the procedure will be compared to the standard 12-lead ECG employed for the diagnosis of inferior wall myocardial infarction. The diagnostic criteria of inferior wall myocardial infarction in the ECG will be considered as new ST-elevation at the J-point in 2 leads among II, III, aVF leads, with the cut-point of ≥1 mm (Figure 3). The resting ECG results of each method will also be compared. Wired 6-lead monitor recordings will be further used to verify the handheld 6-lead ECG recordings.

### 2.6. Sample Size Estimates

Based on the diagnosis of inferior wall myocardial infarction using a standard 12-lead ECG, it was assumed that >98% of inferior wall myocardial infarctions can be diagnosed using a handheld 6-lead ECG. Based on the existing literature, we assumed that the prevalence of inferior wall STEMI among all STEMI patients would be 40%, and subjects with STEMI and those without STEMI would be recruited at a 1:2 ratio [13]. To obtain the Cohen’s kappa coefficient, with minimum acceptable kappa (κ0): 0.8, expected kappa (κ1): 0.98, proportion of outcome (p): 0.4 × 1/3 = 0.13, significance level (α): 0.05 (two-tailed), power (1 − β): 80%, expected dropout rate: 10%, the target number of subjects was deemed to be 216 in total (72 subjects with STEMI and 144 subjects without STEMI).

### 2.7. Statistical Analysis

For continuous variables, the mean or median with standard deviation or interquartile range will be presented. The t-test or one-way ANOVA will be performed for data that follow a normal distribution, depending on the normality test results obtained by the Shapiro–Wilk test; otherwise, the Mann–Whitney U or Kruskal–Wallis test will be performed. Categorical variables will be presented as frequencies and percentages and will be compared using the chi-square test and Fisher’s exact test. The relationship between variables will be assessed using Pearson’s or Spearman’s correlation coefficients. The importance of each variable will be analyzed using logistic regression analysis. The Cohen’s kappa coefficient test will be performed to confirm the diagnostic concordance between each test; further, the concordance rate, rate of positive agreement, and McNemar’s test will be also performed. The sensitivity and specificity of the 6-lead ECG will be assessed based on the evaluation results obtained by the standard 12-lead ECG. To assess the sensitivity and specificity of the 6-lead handheld ECG in detecting inferior wall STEMI compared to the 12-lead ECG, comparisons between these two types of ECGs will be conducted on patients with various types of STEMI as well as on patients presenting with chest pain without STEMI. This analysis aims to determine whether the 6-lead handheld ECG performs comparably to the gold standard 12-lead ECG in identifying inferior wall STEMI among patients presenting with chest pain. Additionally, to evaluate the consistency of the 6-lead handheld ECG across different patient positions, both sitting and supine positions will be utilized to perform handheld ECGs on all STEMI patients and those presenting in outpatient settings. This analysis aims to demonstrate minimal variation in the performance of the 6-lead handheld ECG based on patient posture (Figure 2).

The Statistical Package for the Social Sciences (SPSS version 26.0; IBM SPSS Statistics, Armonk, New York, NY, USA), R (version 4.4.1; R Core Team, 2022) and RStudio (version 2024.04.2+764; Rstudio Team, 2022) will be used for statistical analyses. Statistical significance will be set at *p* < 0.05.

### 2.8. Current Status

The HINT-MI study planned to complete a 1-year enrolment period for the 216 pre-specified participants. The first participant was enrolled in December 2022, and approximately 114 patients, including 24 patients with STEMI, were enrolled by the end of May 2024. Enrolment may be completed in late 2024, and the primary results of the HINT-MI study will be available by early or mid-2025.

### 2.9. Ethical Conduct

The study protocol was approved by the independent ethics committee of Ewha Womans University Mokdong Hospital, Seoul, Korea (EUMC 2022-09-034-05), and all participating centers obtained approval from their corresponding ethics committees. All study procedures will comply with the principles of Good Clinical Practice and the 1975 Declaration of Helsinki. Only patients who provide written informed consent after receiving sufficient explanation will be included.

## 3. Discussion

As new handheld or wearable ECG devices have been developed, and smartwatch ECG recordings have become possible, several studies have been conducted to determine whether STEMI can be diagnosed using mobile devices. A study was conducted to determine whether STEMI can be diagnosed by serial recording using a smartphone and a single-lead ECG device. In this study, the ECG was performed by placing an electrode connected to the device using an electric wire with electrode clamps on an ECG sticker attached to each lead [9]. In another study, an ECG was recorded by serial contact between the smartwatch and the ECG electrode area, and ST wave changes were confirmed with a Cohen κ coefficient, 0.88; 95% CI, 0.78–0.97 [10].

A six-lead recorder-type ECG device based on a smartphone (AliveCor KardiaMobile 6 L), a small device (9.0 × 3.0 × 0.72 cm) that can measure and transmit the ECG, has been developed. This mobile 6-lead handheld ECG recording device was compared with a 12-lead ECG to validate interval duration measurements such as heart rate, PR interval, QRS duration, and QT interval [14]. While a previously described study used a single-lead ECG device, Mercer et al. reported methods employing a handheld 6-lead ECG recorder and ECG patches simultaneously to record chest lead equivalents [8].

Although these studies have shown the possibility of using mobile ECG recordings for STEMI, the methods used in these previous studies were more time consuming than a conventional ECG. Because rapid diagnosis is important for improving prognosis in STEMI [2], our study focuses on reducing the time required for diagnosis. To address this need for speed, we have prioritized investigating the rapid diagnosis of inferior wall STEMI. This particular focus is because diagnosing inferior wall STEMI requires only six leads, aligning with our objective to expedite diagnosis using a handheld 6-lead ECG device.

Another factor to consider is that the 6-lead handheld ECG device can record while an individual is sitting or lying down, depending on the convenience of use. We will also examine whether there is a difference between the sitting and supine positions when recording with a six-lead ECG device and determine whether it is suitable to use the six-lead ECG recorded in the supine position for diagnosis. In addition, since the results of each ECG method will be evaluated blindly, bias related to the reading itself will be reduced.

Through this study process, confirming the ability to rapidly diagnose inferior STEMI could serve as a foundation for future advancements, such as using artificial intelligence (AI) to generate 12-lead ECGs from 3-lead ECGs for diagnosing all types of STEMI. AI research in this area is currently being published as a preprint and is expected to further develop in the future [15].

This study aims to demonstrate the compatibility of the six-lead ECG recording device for the rapid diagnosis of inferior wall MI compared to the 12-lead standard ECG. By potentially reducing the time required for diagnosing inferior wall STEMI, physicians would have more opportunities to improve outcomes for patients with STEMI.

### Study Limitations

In this study, we will compare two types of ECG for the diagnostic accuracy of inferior wall STEMI: a 6-lead ECG using a handheld device and a standard 12-lead ECG. This study will have some limitations. The sets of ECGs will not be recorded simultaneously. Owing to the variability in autonomic tone and heart rate, it is not expected that the measurements taken 5–15 min apart will be identical. This limits the usefulness of these data for comparing the precision of ECG measurements obtained using the two ECG recording methods. However, the diagnosis of STEMI by ECG is primarily based on the elevation of the ST wave; therefore, it is unlikely that these timing differences will significantly impact the overall diagnosis of STEMI. Additionally, diagnosing other types of STEMI, such as anterior and lateral STEMIs, requires additional precordial leads beyond the six available in our handheld device. Nevertheless, prompt diagnosis of inferior wall STEMI could potentially lead to earlier interventions and improved patient outcomes.

## 4. Conclusions

If a handheld 6-lead ECG device is capable of diagnosing inferior wall STEMI, it can reduce the time required to diagnose inferior wall STEMI and enable early treatment.

## Data Availability

No new data were created or analyzed in this study. Data sharing is not applicable to this article.

## Abbreviations

ECG, electrocardiography; PCI, percutaneous coronary intervention; STEMI, ST-T segment elevation myocardial infarction; and HINT-MI, handheld 6-lead ECG device for early detection of acute inferior wall ST-T segment elevation myocardial infarction.
